# Algal origins of core land plant stress response subnetworks

**DOI:** 10.1111/tpj.70291

**Published:** 2025-06-23

**Authors:** Armin Dadras, Pauline Duminil, Sophie de Vries, Iker Irisarri, Ivo Feussner, Jan de Vries

**Affiliations:** ^1^ Department of Applied Bioinformatics, Institute of Microbiology and Genetics University of Goettingen Goldschmidtstr. 1 Goettingen 37077 Germany; ^2^ Department of Plant Biochemistry, Albrecht von Haller Institute for Plant Sciences University of Goettingen Justus‐von‐Liebig Weg 11 Goettingen 37077 Germany; ^3^ Department of Biodiversity and Evolutionary Biology Museo Nacional de Ciencias Naturales (MNCN‐CSIC) c/ José Gutiérrez Abascal 2 Madrid 28006 Spain; ^4^ Department of Plant Biochemistry, Goettingen Center for Molecular Biosciences (GZMB) University of Goettingen Justus‐von‐Liebig Weg 11 Goettingen 37077 Germany; ^5^ Department of Applied Bioinformatics, Goettingen Center for Molecular Biosciences (GZMB) University of Goettingen Goldschmidtstr. 1 Goettingen 37077 Germany; ^6^ Department of Applied Bioinformatics, Campus Institute Data Science (CIDAS) University of Goettingen Goldschmidtstr. 1 Goettingen 37077 Germany

**Keywords:** plant evolution, stress physiology, co‐expression networks, plant terrestrialization, streptophyte algae

## Abstract

We computed co‐expression networks from more than 2200 samples of nine species across 600 million years of divergent streptophyte evolution and infer that the streptophyte algal ancestors of land plants already had a remarkable fraction of the embryophytic stress response system. Despite its phytohormone‐independent origin, homologs of all core components of the drought hormone abscisic acid (ABA) subnetwork are present, and we find that most are co‐expressed in streptophyte algae and land plants; this subnetwork was thus co‐opted in embryophytes by bringing it under the regime of ABA. The last common ancestor of embryophytes and Zygnematophyceae algae had ancient stress‐responsive pathways, enabling it to face the stresses typical of the land environment – even before the origin of land plants – while evolution on land led to the adaptive refinement of these responses.

## Significance Statement

Dadras et al. computed co‐expression from over 2200 samples of nine species, identified the shared stress network across 600 million years of divergent streptophyte evolution; in that shared network and despite its phytohormone‐independent origin, core components of the abscisic acid (ABA) subnetwork are co‐expressed in streptophyte algae and land plants.

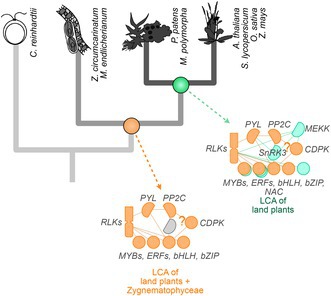

The evolutionary origin of complex stress signaling cascades in land plants is a major question in the field. Here, we computed co‐expression from over 2200 samples of nine species, identified the shared stress network across 600 million years of divergent streptophyte evolution, and provided all through an interactive interface to the community.

## INTRODUCTION

Plant terrestrialization is one of the great singularities in evolution; it yielded the Embryophyta (land plants) (de Vries & Archibald, 2018). One key aspect of plant terrestrialization was adequately withstanding the range of terrestrial stressors (Fürst‐Jansen et al., 2020). To understand what transpired during the conquest of land by plants, we must compare land plants to their closest relatives. Phylogenomics have clarified that the Zygnematophyceae are the algal sister lineage to land plants (One Thousand Plant Transcriptomes Initiative, 2019; Wickett et al., 2014); the class Zygnematophyceae has deeply diversified into five orders and includes unicellular and multicellular algae (Hess et al., 2022). The genomes of multiple representatives of streptophyte algae and land plants have been sequenced in recent years and paved the path to comparative genomics building on fine‐combed phylogenomics (Becker et al., 2020; Bierenbroodspot, Darienko, et al., 2024; Bierenbroodspot, Pröschold, et al., 2024; Cheng et al., 2019; Irisarri et al., 2021; Kunz et al., 2025; Li et al., 2018; Nishiyama et al., 2018; Sekimoto et al., 2023). This way, the presence and absence patterns of relevant genes have been pinpointed. These include several genes coding for protein homologs that act in important phytohormone signaling pathways such as the growth hormone auxin (Carrillo‐Carrasco et al., 2023; Flores‐Sandoval et al., 2018; Kuhn et al., 2024; Mutte et al., 2018) and the stress hormones ethylene (Ju et al., 2015; Van de Poel et al., 2016; Van de Poel & de Vries, 2023) and abscisic acid (Cheng et al., 2019; de Vries et al., 2018). But overall the question of how hormonal signaling networks evolved remains wide open (Blazquez et al., 2020; Briones‐Moreno et al., 2023; Hernandez‐Garcia et al., 2021; Zegers et al., 2024), despite diverse the fact that routes for specialized metabolites that could have signaling (next to other) functions have also been detected in streptophyte algae (Fernie et al., 2024; Jiao et al., 2020; Maeda & Fernie, 2021; Rieseberg et al., 2023) alongside conserved downstream cell biological outputs (Cuming et al., 2007; Vilarrasa‐Blasi et al., 2024; Zegers et al., 2025). Thanks to efforts by the research community on plant evolution, this inferred potential genetic basis constituting a stress physiological program shared between land plants and streptophyte algae is constantly growing. Owing to the advancements thus made, we can now globally study their responsiveness to abiotic stressors.

The critical thresholds in RNAseq sample numbers for comparative co‐expression network analysis have been surpassed for a phylodiverse set of streptophyte algae and model embryophytes (Dadras et al., 2023; Feng et al., 2024; Fernandez‐Pozo et al., 2020; Rieseberg et al., 2025; Tan et al., 2023), previously limited to species‐specific co‐expression network analyses (Ruprecht et al., 2017), and expression profile analysis for multiple species (Proost & Mutwil, 2018). For example, recently Granger causality combined with random forest were used to predict a gene regulatory network of three 600‐million‐year‐divergent streptophytes (Rieseberg et al., 2025).

Gaussian Graphical Models (GGMs) are used to create biological networks such as co‐expression networks based on gene expression data (Wang et al., 2016; Zhao & Duan, 2019). Co‐expression networks depict genes as nodes and conditional relationships as edges, with the existence or non‐existence of a connection defined by the corresponding element in the estimated precision matrix. GGMs use a multivariate normal distribution to model gene expression profiles and calculate co‐expression associations. To infer a co‐expression network with GGMs, we assume a multivariate normal distribution between the expression profiles of a set of genes. The estimate of the inverse of the covariance matrix (precision matrix) is then examined to find which pairs of genes have significant conditional dependence, and the co‐expression network is constructed based on the dependence structure. The nonzero off‐diagonal elements of the estimated precision matrix represent edges in the network. In most biological experiments, the number of genes is much larger than the number of samples. Therefore, researchers should use some form of regularization to induce sparsity in the estimation of the p‐dimensional precision matrix.

Regularization methods are used to estimate sparse precision matrices, as gene expression data is often high‐dimensional and sample numbers are limited (Friedman et al., 2008). Graphical Lasso (GL) is a widely used method that utilizes L1 regularization to induce sparsity in network reconstruction (Danaher et al., 2014; Lyu et al., 2018; Seal et al., 2023). In our multi‐condition gene co‐expression study, co‐expression profiles across multiple conditions are available, and it is of great interest to find out how similar or dissimilar the co‐expression networks are across these conditions. Pairwise comparison is limited. A joint analysis of co‐expression networks harnessing shared information across different conditions can be significantly more powerful than individual analyses. Fused Graphical Lasso (FGL) estimates multiple GGMs simultaneously while promoting similarity between networks and increasing the statistical power of analysis through borrowing information across conditions (Danaher et al., 2014). However, FGL operates under the assumption of identical resemblance across all conditions, which can be limiting.

The standard LASSO penalty encourages sparsity in the network estimation, and the pairwise fused LASSO penalty ensures that the network shares some degree of similarity. The fused LASSO penalties inherently assume that the precision matrices, and consequently, the co‐expression networks, in all conditions are equally similar to each other. This assumption is rigid and may easily be violated in most real data scenarios. To account for this condition (similarity and dissimilarity) in an FGL framework, Condition Adaptive Fused Graphical LASSO (CFGL) has been developed. The CFGL method includes condition‐specific weights to consider different levels of similarity among networks based on the studied data and improves the accuracy of predictions (Lyu et al., 2018). The penalty term considered in CFGL is a modification of the pairwise fused LASSO penalty that incorporates binary weights matrices capturing condition‐specificity. Upon estimating a precision matrix under condition *k*, the gene co‐expression network would be constructed by representing genes as nodes and conditional dependencies as edges in a graph. To be more specific, two genes *i* and *j* under a given condition will only be connected in the graph if and only if the corresponding element in the precision matrix is not zero. However, in practice, this method is only feasible for analyzing a few genes (~1000) and a maximum of three conditions. Rapid Condition Adaptive Fused Graphical Lasso (RCFGL) was developed to solve these problems without losing precision by combining the ideas of CFGL and Fused Multiple Graphical Lasso (FMGL) (Yang et al., 2015) and calculates sequential condition‐specific fused lasso penalties across the conditions instead of the pairwise penalty (Seal et al., 2023).

**Figure 1:**
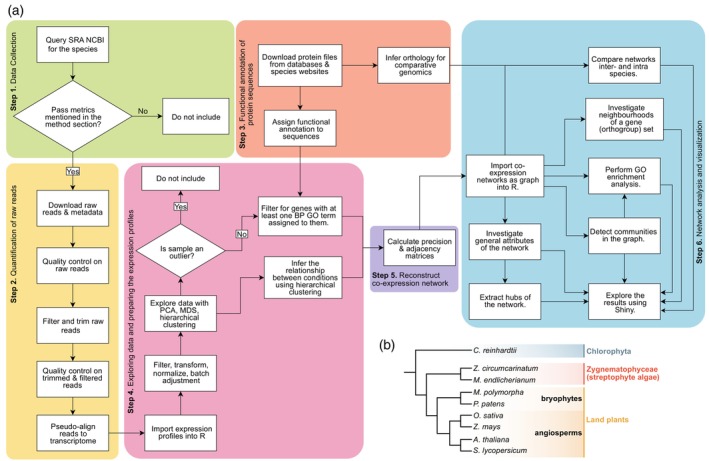
Setup of the study. (a) The schematic bioinformatic workflow and (b) cladogram of species under study and the groups they represent.

Here, we studied the conserved and divergent influence of stress on co‐expression networks, depicting genes as nodes and conditional relationships as edges defined by the corresponding element in the estimated precision matrix. We consolidated several methods and systematically gathered RNAseq reads from public databases and quantified them using the most updated gene models (Figure 1a). Since RCFGL was developed to work on >1000 genes and several conditions without losing precision (Yang et al., 2015), we used this state‐of‐the‐art method to investigate networks across 15 conditions and 9 species. Our approach recovers predictions that align with functionally confirmed knowledge while yielding evolutionary insights: our comparisons of networks pinpoint to the ancient origins of several concerted actions in genes for key stress response functions of land plants. Foremost, we find that the genes constituting the ABA core‐signaling module co‐express in land plants and their closest algal relatives. We therefore infer that the genes that have assembled into the ABA core signaling module likely co‐expressed in their common ancestor – probably before playing a phytohormone‐dependent role (Sun et al., 2019) – within a subnetwork rich in cross connections to key stress response pathways.

All results are available at the StreptoNet Shiny app: https://rshiny.gwdg.de/apps/streptonet/.

### A co‐expression framework from more than 2200 samples across 600 million years of streptophyte evolution

We downloaded RNAseq data derived from stress treatments of seven land plants, two zygnematophytes (the closest algal relatives of land plants), and the chlorophyte *Chlamydomonas reinhardtii* (Figure 1b). After filtering for quality (see “Methods” section), a total of 2279 RNAseq samples were included in our study (Table S1). Naturally, *A. thaliana* had the largest sample proportion, with *O. sativa* coming in second; *C. reinhardtii* and *Marchantia polymorpha* had the lowest numbers of samples (27 and 48 samples, respectively). Hence, the networks of these two latter species were constructed on less robust foundations. Nevertheless, their inclusion allows several evolutionary‐relevant inferences.

We pre‐filtered the input for genes that had at least one Gene Ontology (GO) biological process annotation and were expressed in our samples to ensure calculability of the networks; the runtime of RCFGL, despite being significantly faster than CFGL, is still proportional to the number of conditions (*k*) and the squared number of genes (*p*) $\left(O\left(p^{2}k\right)\right)$. The input ranged from 4560 genes (*C. reinhardtii*) to 11 308 (*Zea mays*) (Table S2; average of ~31% of genes in the genomes) and 4–11 conditions (Table S3). On balance, in most cases a minimum of 20 samples per condition were required to obtain a high‐quality co‐expression network (Tables S4–S8; for the conditions investigated see Tables S9–S17). For instance, the networks of *C. reinhardtii* or the ABA network of *M. polymorpha* – the two species with the lowest number of conditions – exhibited a lower number of edges, nodes, and components, compared to networks from larger datasets. Eventually a saturation threshold is reached, causing the number of connections and components to plateau beyond a certain point, exemplified by the *O. sativa* samples.

All data presented here are available through StreptoNet, a Shiny app that allows various network investigations for the nine representatives of streptophytes, allowing the comparison of networks across 600 million years of evolution.

### Co‐expression network predictions align with functionally characterized molecular connections under stress

To scrutinize whether our approach recovers meaningful biological programs, we examined the networks that were constructed for *A. thaliana* under UV treatment to compare the predictions of our model with what is known from the literature. Our UV network contains 6027 genes with 11 808 connections, with an average of 85 components per connection. The largest hub in the network (the gene with most edges) connected 5830 genes. The top 50 hubs included transcription factors like the master regulators (Cruz‐Ramirez et al., 2012; Shahan et al., 2022) Scarecrow‐like, the *MYB12* member *PRODUCTION OF FLAVONOL GLYCOSIDES 1* key for the synthesis of UV‐shielding flavonoids (Stracke et al., 2010), the light signaling regulator *CRYPTOCHROME‐INTERACTING BASIC‐HELIX–LOOP–HELIX 1* (Liu et al., 2008), and the photooxidative scavenger *EARLY LIGHT‐INDUCED PROTEIN* (*ELIP*) (Hutin et al., 2003) (Table S18). GO terms recovered meaningful genetic cohorts associated with response to UV and ROS, and specialized metabolism (Table S19; Figure S1a). Scrutinizing neighborhoods of genes with the TAIR keyword ‘response to UV’ (Figure S1b) pinpointed genes related to photosynthesis and light reactions, biosynthesis of flavonoid and phenylpropanoids (including the key enzyme‐coding genes *PHENYLALANINE AMMONIA LYASE* (Cochrane et al., 2004; Dixon & Barros, 2019; Jun et al., 2018), 4‐*COUMARATE–COA LIGASE* (*4CL*) (Costa et al., 2005; Hu et al., 2010), and more), signaling and general response to stress [e.g., *PEROXIDASE* (Haslekås et al., 2003)], modifiers of cell wall [e.g., *EXPANSIN* (Cosgrove, 2000; Sampedro & Cosgrove, 2005; Tenhaken, 2014)], hormone biosynthesis and signaling [e.g., *CYTOKININ OXIDASE/DEHYDROGENASE* (Schmulling et al., 2003; Werner et al., 2003)], and multiple well‐known transcription factors (Table S20).

Similarly, the *P. patens* network related to wounding and jasmonate response contains 2503 genes that have 4166 connections and an average of 14 connected components. The biggest cohort of connections contained 2475 genes and highly connected hubs were genes for transcription factors such as *YIN YANG 1* (*YY1*) (Li et al., 2016) and *HOMEOBOX PROTEIN* (*HB*) (Söderman et al., 1996), and ABA signaling modulators such as *MOTHER OF FT AND TFL1* (*MFT*) (Xi et al., 2010); while the function of MFT in *Physcomitrium* is elusive, its key regulator, the *Physcomitrium* homologs of DELAY OF GERMINATION 1 (DOG1), forms a regulatory network with conserved function (Vollmeister et al., 2024). Furthermore, they included *BETA GLUCOSIDASE 18* (Lee et al., 2006), MAP kinase genes such as *MAPKKK* (Wang et al., 2007), which are also stress hubs in *Physcomitrium* (Rieseberg et al., 2025; Toriyama et al., 2022), photoprotection such as *ELIP* (Benning, 1998; Hutin et al., 2003), and members of biosynthesis of specialized metabolism such as *PAL* and *THI* (Papini‐Terzi et al., 2003) which is in line with the finding that biotic stressors induces expression of almost all phenylpropanoid biosynthesis‐relevant enzyme‐coding genes in *P. patens* (Reboledo et al., 2021). This is further underpinned by cell wall modification genes like xyloglucan endotransglucosylase/hydrolases (*XTH*) (Nishitani & Vissenberg, 2006), *α‐XYLOSIDASE1* (Sampedro et al., 2010), and *CELLULOSE SYNTHASE* (*CES*) (Richmond & Somerville, 2000), receptors such as *ROP BINDING PROTEIN KINASE 1* (Molendijk et al., 2008), *GLYCEROL‐3‐PHOSPHATE ACYLTRANSFERASE* key for forming the stress‐warding cuticle of *Physcomitrium* (Lee et al., 2020), and genes relevant upon oxidative and heat stress, with aligning GO terms (Figure S1c; Tables S21 and S22).

Guilt‐by‐association via co‐expression yielded 84 *P. patens* genes that are in the same hierarchical ortholog group (HOG) as *A. thaliana* genes for jasmonate biosynthetic processes, including dozens of MYBs [regulating various genes salient to the biosynthesis of phenylpropanoid‐derived compounds like flavonoids, lignin, and other diverse specialized metabolic and developmental processes (Dubos et al., 2010; Liu et al., 2015)], WRKY transcription factors [involved in stress and defense responses (Jiang et al., 2017)], and more (Table S23). Yet, multiple genes in the *P. patens* genome are not assigned to a HOG containing *A. thaliana* genes, possibly pointing to divergent response tracks. These results highlight that while with a higher the number of samples the network becomes more robust, even with a smaller number of samples (such as *P. patens* jasmonate/wounding having only nine samples) we can get predictions that are in agreement with biological expectations.

Together, these data point to a reliable recovery of stress networks. Since heat stress has been ubiquitously applied to all the selected species, we used it as model to infer conserved stress response along the phylogeny of streptophytes by mapping shared HOGs onto our co‐expression networks across species. We investigated HOGs whose connections either (i) form or (ii) dissolve under heat treatment. Hubs that emerge under heat are genes coding for HEAT SHOCK PROTEINs (HSPs) and PEROXIDASE (PER/PRX) active in ROS homeostasis (Garcia‐Caparros et al., 2021); signaling‐relevant genes for ETHYLENE RESPONSE FACTOR (ERF) (Huang et al., 2021, 2023; Larkindale & Knight, 2002; Muller & Munne‐Bosch, 2015), cell wall‐relevant genes such as XTHs (Van Sandt et al., 2007) and expansins (Cosgrove, 2015); along these went enriched GO terms salient to heat stress and more (Tables S24 and S25). Vice versa (Control‐Heat), we recovered 19 427 shared associations and 5075 HOGs. Many hubs are shared in this network and the one discussed above (Table S26), which means some of these genes are balancing regulators – but some hubs were specific to one network, for example, *PAL* (Tables S27–S29).

### A union network across 600 million years of streptophyte evolution

Co‐expression networks reflect dynamic associations between genes. Genes co‐expressed under a given stress might not be associated under another. The dynamic nature of co‐expression networks poses challenges when conducting large‐scale comparisons involving multiple conditions or comparisons across different species and treatments. Thus, we employed the union of condition‐specific networks for each species to decrease the number of networks to be compared from 55 condition‐specific networks of all species to nine species‐specific networks with various GO terms related to stress response, cell wall remodeling, signaling processes upon external and internal cues, and specialized metabolism (Figure S2). While the capture of these GO terms suggests potentially interesting lineage‐specific patterns, it primarily demonstrates that our union network approach effectively captured meaningful cohorts associated with environmental responses (Figure S2).

**Figure 2:**
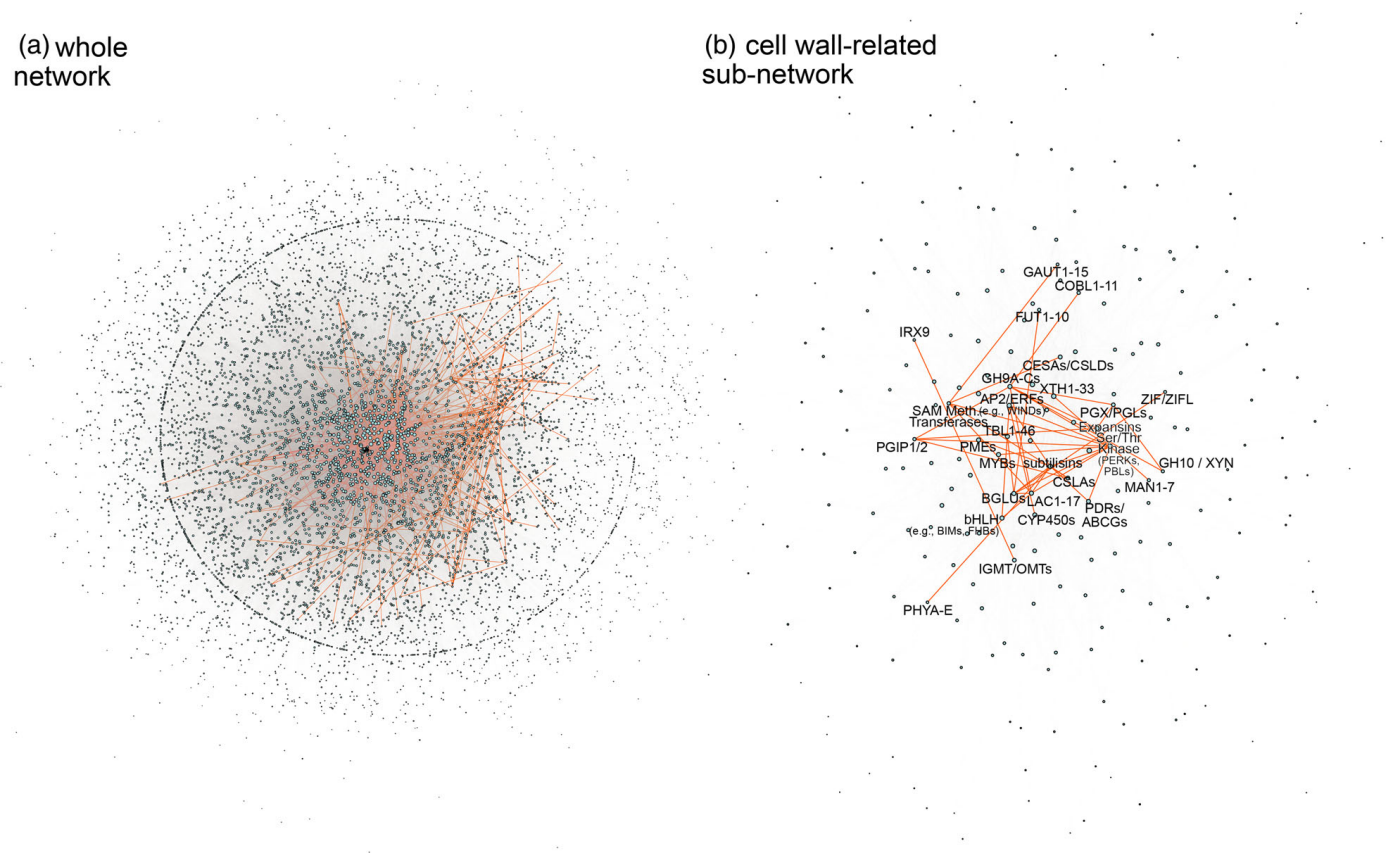
Aggregated networks of conserved connections among land plants and streptophyte algae. (a) Complete network; (b) subnetwork filtered for cell wall orthogroups (based on GO terms). Names were assigned to each orthogroup based on the *Arabidopsis thaliana* genes that were present in that orthogroup in all subnetworks.

To extract the core stress response network of all streptophytes, we aggregated all species networks of 952 to 5305 nodes and 1909 to 57 016 edges into a union network of 7353 nodes connected by 154 468 edges. From this, we extracted a robust conserved biological subnetwork that included 407 nodes connected by 579 edges that we infer as shared across the 600‐million‐year‐divergent streptophytes (Figure 2a). While we focus here on the shared core, our method highlighted new players of stress response pathways in (non‐)model organisms, such as an extensive Zygnematophyceae‐specific subnetwork. The detectability of meaningful biological programs in this streptophyte union network is exemplified by the discovery of modules for conserved processes like cell wall biogenesis (Figure 2b), which is well‐known from co‐expression analyses (Ali et al., 2025; Proost & Mutwil, 2017; Sibout et al., 2017). These included *CES* and *GLYCOSIDE HYDROLASE* genes as well as genes known from lignified cell wall formation such as diverse genes coding for methyltransferases, suggesting that indeed the regulatory network for lignification could be ancient (see Dadras et al., 2025). Interestingly, also other genes that we previously highlighted in co‐expression network analyses of Zygnematophyceae and land plants (Dadras et al., 2023; Rieseberg et al., 2025) were recovered, including *COBRA* (Ko et al., 2006; Roudier et al., 2002; Schindelman et al., 2001). Yet, also specific and adaptive modules were recovered – to which we turn next.

### ABA‐regulated processes plug onto an ancient co‐expressed module

**Figure 3:**
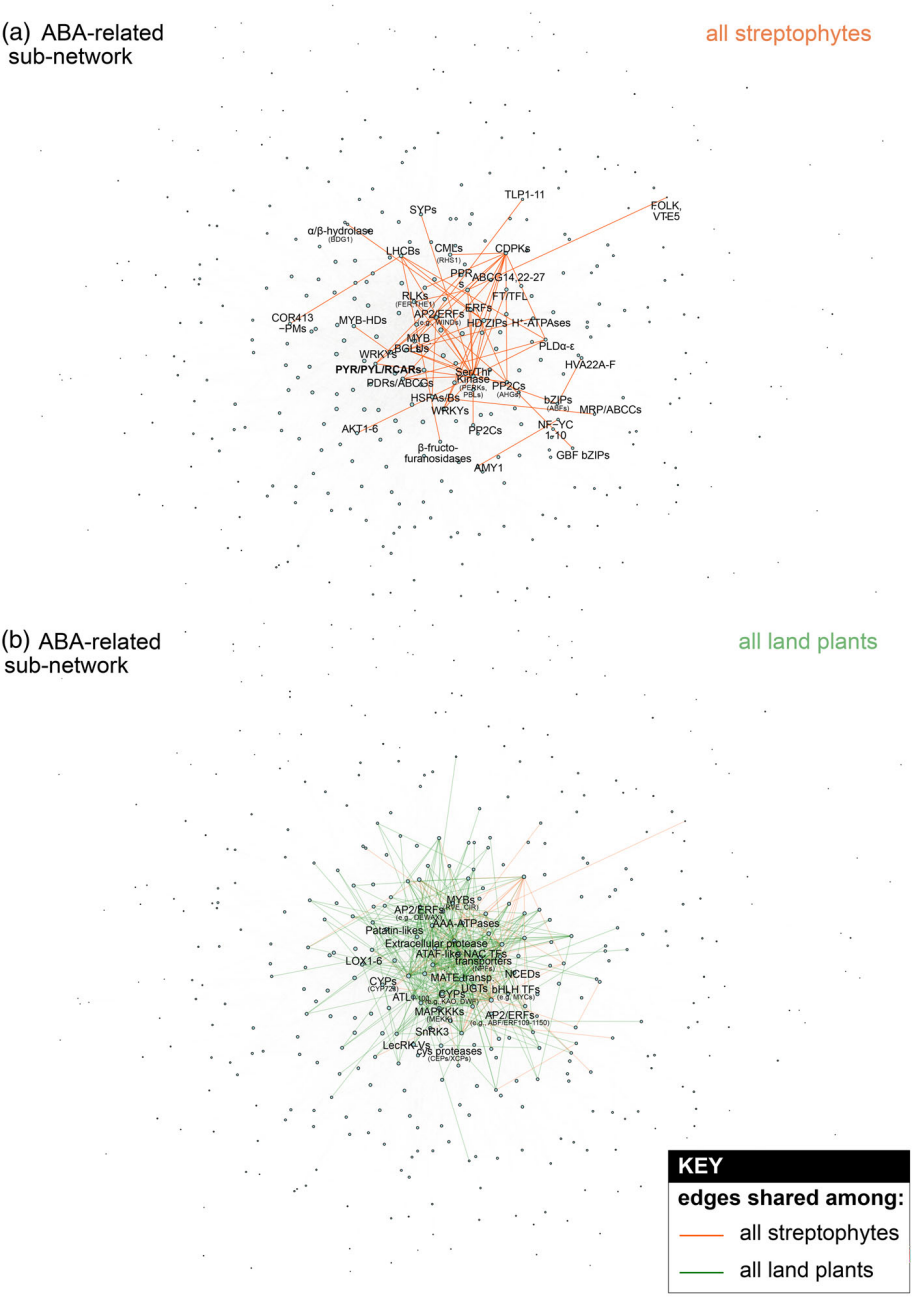
Aggregated subnetwork of conserved connections among land plants and streptophyte algae salient to abscisic acid signaling. (a) Subnetwork filtered for orthogroups related to abscisic acid (based on GO terms). The associations that were present with at least one tracheophyte, one bryophyte, and one zygnematophyceae algae were depicted as conserved among the last common ancestor of land plants and streptophyte algae via orange. (b) Similar to (a) but with the edges that were present only in land plants (i.e., at least one tracheophyte and one bryophyte) shown in green. The names of the top 20 most connected gene hubs that were not present in (a) (i.e., emerging hubs) were labeled.

Land plants use an intricate molecular network to respond to abiotic stress (Scheres & van der Putten, 2017; Waadt et al., 2022). Genes of famous stress response pathways are present (at least partially) in the closest algal relatives of land plants (Cheng et al., 2019; de Vries et al., 2018; Hori et al., 2014; Kunz et al., 2025; Nishiyama et al., 2018). Yet, even if we compare the within‐embryophyte networks of a moss and an angiosperm, we noticed that conserved connections are just a very small portion of the whole network. Among the more than 150 000 edges, 3659 edges are shared between at least one angiosperm and one bryophyte, while 676 edges are shared between at least one angiosperm and one Zygnematophyceae. Additionally, 361 edges are shared between at least one bryophyte and one Zygnematophyceae. Within the groups, 118 924 edges are shared exclusively among angiosperms, 34 035 edges are shared exclusively among bryophytes, and 8320 edges are shared exclusively among Zygnematophyceae. During streptophyte macroevolution, the same components thus appear integrated into different biological modules and as such are likely employed in slightly different manners (Goldbecker & de Vries, 2025; Zegers et al., 2024). Nonetheless, some subnetworks appear robust during streptophyte evolution. Here, the subnetwork that particularly caught our attention due to its early and robust elaboration in streptophyte evolution relates to abscisic acid (Figure 3a,b).

Novel gene regulators are a prime source of biological innovation. Most genomic evolution in land plants builds on established regulatory cassettes that are re‐shuffled and give rise to novelty (Romani & Moreno, 2021; Zegers et al., 2024). A prime example that has emerged over the last years is multicellularity: it is notoriously difficult to pinpoint genes specifically involved in multicellularity. This is probably because it is not novel genes that do the trick but novel regulation (Bierenbroodspot, Darienko, et al., 2024; Prochnik et al., 2010; Umen, 2014; Umen & Herron, 2021). In plants, phytohormones govern many processes and thus their evolution is linked to novelties in physiology, growth, and development, and more (Blazquez et al., 2020; Hernandez‐Garcia et al., 2021). Yet, many of them are present in streptophyte algae, and so are (at least in part) their biosynthesis and signaling components (Rieseberg et al., 2023). Thus, adding streptophyte algae to comparative approaches can yield insights into the possible landscape of functionalities in a genetic hierarchy actualized by different confirmations – and the (re‐)wiring that occurred through evolution (Goldbecker & de Vries, 2025, Zegers et al., 2024). ABA is here an interesting case.

**Figure 4:**
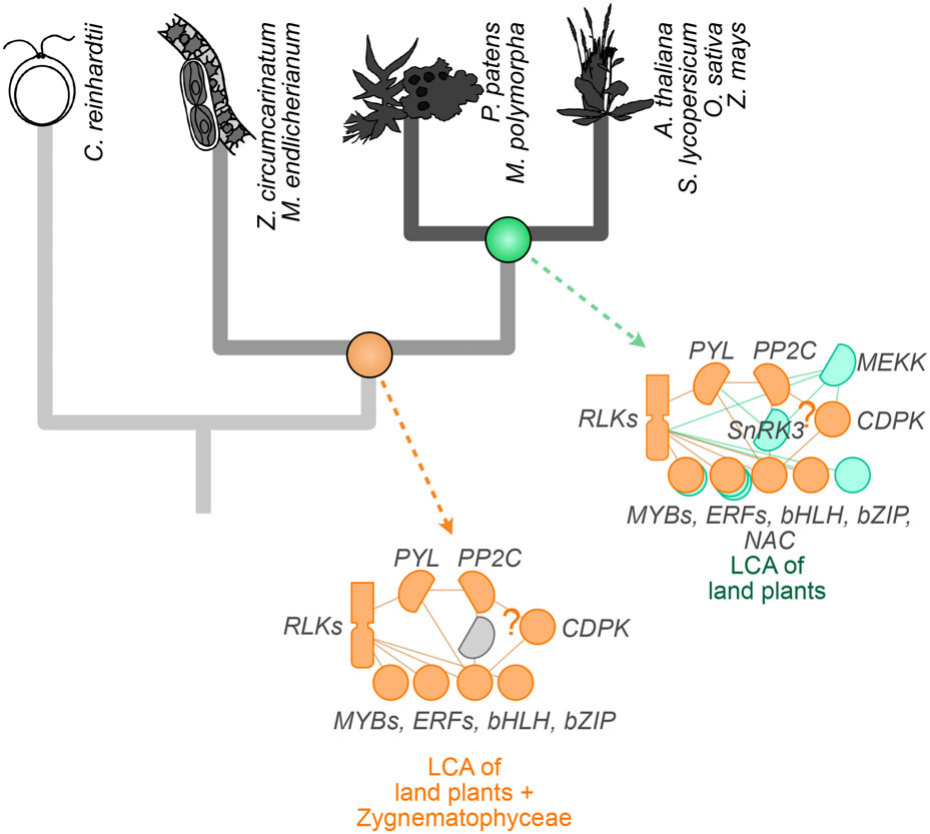
Scenario for the evolution of abiotic stress perception and signaling. A schematic model that summarizes the emergence of stress response actors in the last common ancestor of land plants and zygnematophyceaen, as well as novel connections and recruitment that occurred after the divergence of land plants and zygnematophyceaen algae.

The core protein cascade for ABA signaling consists of a PYRABACTIN RESISTANCE1/PYR1‐LIKE/REGULATORY COMPONENTS OF ABA RECEPTOR (PYR/PYL/RCAR) – PROTEIN PHOSPHATASE 2C (PP2C) co‐receptor complex and the downstream kinase of the subclass III SUCROSE NONFERMENTING 1‐RELATED PROTEIN KINASE 2 (SnRK2s) (Cutler et al., 2010; Melcher et al., 2009; Miyazono et al., 2009; Park et al., 2009; Santiago et al., 2009; Soon et al., 2012; Yin et al., 2009). Their concerted action involved in the response to terrestrial stressors is not only described in tracheophytes but also in bryophyte model systems like *P. patens* (Cuming et al., 2007; Komatsu et al., 2013; Shinozawa et al., 2019; Stevenson et al., 2016; Zimran et al., 2025). The homologous chassis of genes coding for the PYR/PYL/RCAR, PP2C, and SnRK2 proteins was gained in a common ancestor of Zygnematophyceae and land plants (Cheng et al., 2019; de Vries et al., 2018; Feng et al., 2024) – and in this work recovered in the respective orthogroups (e.g., N0.HOG0000779 containing an entire homologous group of the protein‐coding genes At*SnRK2.1* to At*SnRK2.10* and at least one homolog per zygnematophyte species; see Data S1 and our interactive StreptoNet R Shiny app). Functional investigations building on *in vitro* and heterologous approaches have shown that this cascade likely became ABA‐dependent only later, in an early land plant ancestor (Lind et al., 2015; Sun et al., 2019). What does this mean for their co‐expression (considering this as a proxy for concerted action)? The naïve assumption would be that the receptor–co‐receptor formation becoming ABA‐dependent led to the actualization of the signaling network. Our network analyses highlight that a robust co‐expression of not only a minimal gene set but also of the major players *PYL*, *PP2C*, and genes for downstream bZIP transcription factors co‐express in Zygnematophyceae and thus likely in the common ancestor shared with land plants (Figures 3a and 4). What is more, the genes for proteins that are known to cross‐interact with the ABA core module, including *CDPKs* (Edel & Kudla, 2016; Kim et al., 2010; Zhao et al., 2011) and *RLKs* (Osakabe et al., 2005; Shang et al., 2020; Tanaka et al., 2012), also co‐express in the shared core module (Figure 3a). This underpins that the ‘canonical’ ABA signaling is already actualized in the common ancestor of Zygnematophyceae and land plants – yet the ABA‐dependent receptor–co‐receptor formation was likely a missing attribute that was plugged in later in evolution (Sun et al., 2019). Further, in land plants, this network extended by the addition of several major hubs and the duplication of genes within orthogroups and the emergence of links to other signaling networks like the one built on jasmonate via *LOX* and patatin‐likes (Figures 3b and 4).

While we do observe the co‐expression with a type of *SnRK*s appearing in land plants, these are not the *SnRK2*s from the core signaling module (see Cutler et al., 2010) but *SnRK3*s/*CALCINEURIN B‐LIKE‐INTERACTING PROTEIN KINASE*s (*CIPK*s) coding for proteins that are also well established – albeit far less extensively studied – to be targeted by PP2Cs and involved in ABA‐triggered signaling (Batistic et al., 2012; Lan et al., 2011; Lumba et al., 2014). It should be noted that the recovery of *SnRK3* over *SnRK2* must not have any biological implications about the evolutionary trajectory and/or functional relevance but can simply reflect that one group of kinase‐coding genes can be better recovered from transcriptomic profiles. Further, the co‐expression networks highlight the extension to a HOG of genes coding for homologs of the subfamily of MAP3K, the MEKK family, RAF, and ZIK. This includes *ABA‐INSENSITIVE PROTEIN KINASE1* (*AIK1*). AIK1 is a dephosphorylation target of PP2C and has been proposed to modulate the ABA response (Li, Yang, et al., 2017; Yu et al., 2021). Further, another homolog in the HOG, *MAPKKK18*, is key for drought resistance (Li, Cai, et al., 2017) and its expression is modulated by ABA (Zhou et al., 2021).

Only in our networks on land plant data does a co‐expressional link to the carotenoid cleavage gene *NCED* exist (Figure 3b). This is consistent with (i) the ABA pathway being partially conserved (Azar et al., 2025) but resulting in meager levels of ABA (Feng et al., 2024; Schmidt et al., 2024) and (ii), as far as we know, ABA apparently not being the prime signaling molecule in zygnematophytes (Sun et al., 2019, 2020). The “partial” ABA biosynthetic routes likely contribute to the production of other carotenoid (cleavage)‐derived molecules, which have been measured via mass spectrometry in streptophyte algae (Rieseberg et al., 2024, 2025).

Taken together, this means that there was an intricate regulatory network for stress response that used the core building blocks of the ABA network – but this network acted before the dawn of ABA as a signaling molecule and the dawn of plants on land.

## METHODS

### Data collection and cleaning

The first step of our analysis was to gather a comprehensive set of RNAseq raw reads and quantify the expression of genes for each sample. In order to do that, we utilized a pipeline that consists of FastQC (v0.12.1) (Andrews, 2010), Trimmomatic (v0.39) (Bolger et al., 2014), MultiQC (v1.16) (Ewels et al., 2016), seqtk (v1.4), and Kallisto (v0.48.0) (Bray et al., 2016) implemented via Snakemake (v8.11.3) (Mölder et al., 2021) and available on GitHub (https://github.com/dadrasarmin/rnaseq_quantification_kallisto_pipeline).

We searched SRA NCBI Bioprojects for the following species: *Chlamydomonas reinhardtii*, *Arabidopsis thaliana*, *Mesotaenium endlicherianum*, *Zygnema circumcarinatum*, *Physcomitrium patens*, *Marchantia polymorpha*, *Solanum lycopersicum*, *Zea mays*, *Oryza sativa*, *Selaginella moellendorffii*, *Selaginella tamariscina*, *Selaginella denticulata*, *Selaginella sellowii*, *Selaginella lepidophylla*, *Abies alba*, *Picea abies*, *Pinus koraiensis*, *Larix kaempferi*, *Pinus contorta*, *Picea wilsonii*, *Abies koreana*, *Pinus Massoniana*, *Pinus halepensis*, *Hymenophyllum cruentum*, *Hymenophyllum dentatum*, *Hymenophyllum candiculatum*, *Klebsormidium nitens*, *Klebsormidium dissectum*, *Klebsormidium flaccidum*, *Klebsormidium crenulatum*, *Chlorokybus atmophyticus* [now *C. melkonianii* (Irisarri et al., 2021)], *Mesostigma viride*, *Coleochaete scutata*, *Chlorella vulgaris*, *Dunaliella salina*, *Dunaliella tertiolecta*, *Dunaliella bardawil*, *Ostreococcus lucimarinus*, *Chara braunii*, *Spirogloea muscicola*, *Closterium* sp. *NIES67*, *Anthoceros agrestis*, *Azolla filiculoides*, and *Brachypodium distachyon*.

We were interested in species that have a sequenced genome. We manually investigated all entries and filtered for those who satisfied the following criteria: (i) Sequencing method: RNAseq (no array measurement were included); (ii) wild‐type samples (no mutant samples included); (iii) at least three biological replicates per treatment; (iv) only abiotic stressors such as heat, cold, UV, high light intensity, phytohormone treatment, salt, osmotic, or respective control; (v) photosynthetic tissue; (vi) available experimental design or metadata for each sample in the database; (vii) publicly released datasets; and (viii) bulk RNAseq (no single cell data were included). We mapped the samples of each species to the corresponding transcriptome (derived from the genome annotation) if they had at least 30 samples in total that passed the criteria mentioned above. Based on these criteria, the first nine species listed above were selected. A report of sample sequencing and experimental settings is noted in Table S1.

### Gene expression quantification

In the next step, we went through all quality control reports of filtered and trimmed reads and their mapping reports as follows to keep only samples with high quality and high‐mapping rates for downstream analysis: (i) for each read a minimum leading and trailing quality of 26 in Phred score; (ii) for all reads a minimum sliding window quality of 20 over windows of size 4 for trimming; (iii) minimum read length of 36 base pairs; and (iv) a minimum sequencing depth of 10 million after trimming. Transcriptomes were derived from the following published genome annotations: *A. thaliana* (Cheng et al., 2017), *C. reinhardtii* (Craig et al., 2023), *M. endlicherianum* (Dadras et al., 2023), *M. polymorpha* (V7 from https://marchantia.info/), *Z. circumcarinatum* (Feng et al., 2024), *S. lycopersicum* (Zhou et al., 2022), *O. sativa* (Ouyang et al., 2007), *P. patens* (Bi et al., 2024), and *Z. mays* (Hufford et al., 2021).

### Functional annotation and enrichment analysis

To treat all species similarly and harmonize the functional annotation files for all species, we used eggnog‐mapper (V2.1.12) (Cantalapiedra et al., 2021) and InterProScan (V5.68‐100.0) (Jones et al., 2014) to annotate the protein files of each species. For eggnog‐mapper, we used the following settings: ‐m mmseqs ‐‐itype proteins ‐‐mmseqs_db eggnog_proteins_mmseqs_viridiplantae.mmseqs ‐‐dbmem ‐‐tax_scope 33090 ‐‐go_evidence all ‐‐pfam_realign realign ‐d 33090. For InterProScan, we used the following settings: ‐iprlookup –goterms ‐‐pathways ‐‐seqtype p. Then, we aggregated results from both analyses by merging and removing duplicate rows using Tidyverse (V2.0.0) (Wickham et al., 2019) in R (V4.4.0) (R Core Team, 2024). The Tidyverse package was used for other tasks of data importing, cleaning, wrangling, and exporting in R as well. Enrichment analysis was performed using the clusterProfiler package (V4.12.2) (Wu et al., 2021) with the following settings: pvalueCutoff = 0.05, minGSSize = 10, maxGSSize = 500, qvalueCutoff = 0.05, and all genes that were present in the input file for the co‐expression network calculation as background genes.

### Exploratory data analysis, filtering, transformation, and batch adjustment

To import gene expression into R, we used tximport (V1.32.0) (Soneson et al., 2015) to summarize gene expressions from Kallisto outputs into the gene level by estimating counts from abundance using the “lengthScaledTPM” method. We used the DGEList function from the edgeR package (V4.2.0) (Robinson et al., 2010) to transform the data into log_2_ scale and filter out genes with low expression values – genes with less than 10 counts per million (CPM) in at least 30 percent of samples for each species. Then, we used the qsmooth (V1.20.0) (Hicks et al., 2018) with treatments as a group factor to perform normalization. Next, we used the voom function from the limma package (V3.60.3) (Ritchie et al., 2015) to estimate the mean–variance relationship and prepare gene expression values for linear modeling for each condition and kept the control as our reference condition. The numeric matrix of expression values on the log_2_ scale was extracted and used as input for the empiricalBayesLM function from the WGCNA package (V1.72‐5) (Langfelder & Horvath, 2008). As the author of empiricalBayesLM mentioned, this function preserves variance owing to maintained covariates while removing variation caused by unwanted covariates from high‐dimensional data. Empirical Bayes‐moderated linear regression is used, preferably in a robust (outlier‐resistant) version, to prevent numerical instability. We used covariates such as batch, sequencing instrument, library selection, and sequencing method as unwanted covariates and treatments, temperature, and light intensity as retained covariates. In order to explore the general relationship among our datasets, we performed various exploratory analyses such as principal components analysis (PCA), multidimensional scaling (MDS), and hierarchical clustering using the following functions in R: prcomp, dist, hclust from the stats (V4.4.0) package, plotMDS from the limma package, correlatePCs, pcascree, and plotPCcorrs from the pcaExplorer package (V2.30.0) (Marini & Binder, 2019), and groupOTU from ggtree package (V3.12.0) (Bi et al., 2024). Distance was calculated using the Euclidean method and clustering was done using the complete method. We used genes with at least one GO assignment in the Biological Process (BP) domain as inputs for our co‐expression network analysis and we reported the number of genes that we kept at each step of our analysis in R in Table S2.

### Co‐expression network reconstruction

To construct co‐expression networks, we utilized the RCFGL package (Seal et al., 2023). The number of samples and conditions that were used for each species is reported in Table S4. The order of samples was determined as mentioned in the RCFGL via hierarchical clustering based on the gene expression data. The order of samples for each species was set as follows: (i) *Z. mays*: heat, jasmonates, control, ethylene, drought; (ii) *Z. circumcarinatum*: heat, high light, control, cold, salt; (iii) *S. lycopersicum*: drought, salt, heat, control, cold; (iv) *P. patens*: high light, control, cold, radiation, auxin, jasmonates, heat, salt, ABA, ROS, desiccation; (v) *C. reinhardtii*: UV, acidity, control, heat; (vi) *M. endlicherianum*: cold, control, highlight, heat; (vii) *O. sativa*: cold, acidity, ethylene, control, heat, salt, drought; (viii) *M. polymorpha*: SA, highlight, control, auxin, ABA; (ix) *A. thaliana*: UV, ABA, heat, cold, control, drought, jasmonates, salt, high light. If there was a gene in any species that has no variance, we had to omit it since it is not possible to have features with variance equal to zero in this method. This was executed under Python (V3.10.14). We used the pandas package (McKinney, 2010) for data handling and manipulation in Python and we transformed the input data to have mean 0 using the StandardScaler function of sklearn package (Pedregosa et al., 2011). We performed a grid search as suggested by the authors of the RCFGL package (Seal et al., 2023) for regularization penalty parameters ($\lambda_{1}$ and $\lambda_{2}$) over the following values: 0.01, 0.03, 0.05. These values were obtained from the manuscript of the original work (Seal et al., 2023) as good starting points of search. The best‐fit model was selected as that having the lowest AIC score. The AIC values among other things are reported in Table S3. We used the default values for the ADMM algorithm in this package, which is maximum iteration of 100 and tolerance for the residual convergence of 0.001. We made the adjacency matrices with truncation value of 0.05. We summarized some basic attributes of the co‐expression graphs in Tables S5–S8, such as the number of edges, nodes, number of components for each species and treatments.

### Network comparison using orthogroups

To investigate the co‐expression networks in R, we used the package igraph (V2.0.3) (Csardi & Nepusz, 2006). After importing the networks, we use various functions such as intersection, union, and difference to compare various co‐expression networks and discover conserved and differential patterns across treatments and species. To compare networks across species, we used the orthogroups concept as a proxy for closely related homologs. To infer orthogroups, we used Orthofinder (V2.5.5) (Emms & Kelly, 2015, 2019; Katoh & Standley, 2013; Price et al., 2010) with the following settings: ‐S diamond ‐M msa ‐A mafft ‐T fasttree ‐t 200 ‐a 6 ‐y ‐ft. For additional information on the ABA subnetwork, see the data deposited on Zenodo under 10.5281/zenodo.15591087.

### Shiny app

To build the Shiny app, we utilized a set of different R packages to bring various functionalities into action, and they are as follows: shiny (V1.8.1.1) (Chang et al., 2017), shinydashboard (0.7.2) (Chang & Borges Ribeiro, 2018), tidyverse (V2.0.0) (Wickham et al., 2019), htmltools (V0.5.8.1), DT (V0.33), rBLAST (V1.0.0), Biostrings (V2.72.1) (Pagès et al., 2019), igraph (V2.0.3) (Csardi & Nepusz, 2006), clusterProfiler (V4.12.2) (Wu et al., 2021), visNetwork (V2.1.2), msaR (V0.6.0) (Rauscher & Charlop‐Powers, 2021), and ggtree (V3.12.0) (Xu et al., 2022).

## CONFLICT OF INTEREST

The authors declare no conflicts of interest.

## Supporting information


**Data S1.** All orthofinder results for N0.

**Figure S1.** Cases of *Arabidopsis thaliana* and *Physcomitrium patens* networks that highlight the prediction of characterized concertedly acting genes. Two examples of stress response networks obtained from our approach. The network computed for the responses to UV in *A. thaliana*: (a) enriched GO terms and (b) gene neighborhoods. The network computed for the responses to jasmonate and wounding: (c) enriched GO terms and (d) gene neighborhoods; zoom in highlight interesting modules, showcasing the procedure in the interactive online tool.

**Figure S2.** The percentage of genes with Gene Ontology (GO) terms related to stress across species. Panels show GO terms related to (a) phytohormone, (b) cell signaling, (c) specialized metabolism and cell wall biogenesis. Assignments to the categories (a–c) were carried out based on relevant keywords; redundancy between GO terms in the category is to be expected because phytohormone biology will also be categorized in cell signaling and a specialized metabolism. Species abbreviations: ath, *Arabidopsis* thaliana; cre, *Chlamydomonas reinhardtii*; men, *Mesotaenium endlicherianum*; mpo, *Marchantia polymorpha*; osa, *Oryza sativa*; ppa, *Physcomitrium patens*; sly, *Solanum* lycopersicum; zci, *Zygnema circumcarinatum*; zma, *Zea mays*.

**Table S1.** Summary and metadata of raw reads downloaded from SRA NCBI.
**Table S2.** Summary of total number of genes that survived various steps of analysis.
**Table S3.** Summary of AIC, runtime, number of conditions, and number of input genes used to calculate the co‐expression networks.
**Table S4.** Overview of the number of samples per each condition for each species.
**Table S5.** Total number of edges for the network of each condition in each species.
**Table S6.** Total number of nodes for the network of each condition in each species.
**Table S7.** Total number of components for the network of each condition in each species.
**Table S8.** The size of biggest component for the network of each condition in each species.
**Table S9.** Study design of *A. thaliana* samples investigated in this study.
**Table S10.** Study design of *C. reinhardtii* samples investigated in this study.
**Table S11.** Study design of *M. endlicherianum* samples investigated in this study.
**Table S12.** Study design of *M. polymorpha* samples investigated in this study.
**Table S13.** Study design of *O. sativa* samples investigated in this study.
**Table S14.** Study design of *P. patens* samples investigated in this study.
**Table S15.** Study design of *S. lycopersicum* samples investigated in this study.
**Table S16.** Study design of *Z. circumcarinatum* samples investigated in this study.
**Table S17.** Study design of *Z. mays* samples investigated in this study.
**Table S18.** Top 50 most connected genes in *A. thaliana* network built based on UV samples.
**Table S19.** Table of GO overrepresentation analysis for the network of *A. thaliana* under UV stress.
**Table S20.** Genes connected to the “UV Stress” related genes collected from the UV network of *A. thlaliana*.
**Table S21.** Top 50 most connected genes in *P. patens* network built based on jasmonates and wounding samples.
**Table S22.** Table of GO overrepresentation analysis for the network of *P. patens* under jasmonate and wounding stress.
**Table S23.** Genes connected to the homologs of “jasmonic acid biosynthesis process” from TAIR related genes collected from the jasmonate and wounding network of *P. patens*.
**Table S24.** Top 50 hubs of differential network of heat against control in angiosperm.
**Table S25.** Table of GO overrepresentation analysis for the network of heat‐control from angiosperms.
**Table S26.** Top 50 hubs of differential network of control against heat in angiosperm.
**Table S27.** Top 50 hubs of differential network of heat against control in angiosperms that were not present in the control‐heat network.
**Table S28.** Top 50 hubs of differential network of control against heat in angiosperms that were not present in the heat‐control network.
**Table S29.** Table of GO overrepresentation analysis for the network of control‐heat from angiosperms.
**Table S30.** HOGs that are in the intersection of *A. thaliana* and *O. sativa* heat networks.
**Table S31.** HOGs that are in the intersection of *A. thaliana* and *P. patens* heat networks.
**Table S32.** HOGs that are in the intersection of *A. thaliana* and *M. endlicherianum* heat networks.
**Table S33.** HOGs that are in the intersection of *P. patens* and *M. endlicherianum* heat networks.

## Data Availability

*Code availability*: The scripts used in this project are available at: https://gitlab.gwdg.de/armin.dadras/streptonet_shiny_app. The accession number of all samples that were used are recorded in the study design files in Tables S9–S17. All datasets that were used in this study were downloaded from NCBI SRA. The results can be interactively examined at: https://rshiny.gwdg.de/apps/streptonet/. Additional information can be found on Zenodo under https://doi.org/10.5281/zenodo.15591087 as well as https://doi.org/10.5281/zenodo.13357469 and https://doi.org/10.5281/zenodo.13357538.
