# Supplementary figures and images for: Algal origins of core land plant stress response subnetworks
Source: Plant J. 2025 Jun 23;122(6):e70291. doi: 10.1111/tpj.70291 (PMC12185132; doi:10.1111/tpj.70291)

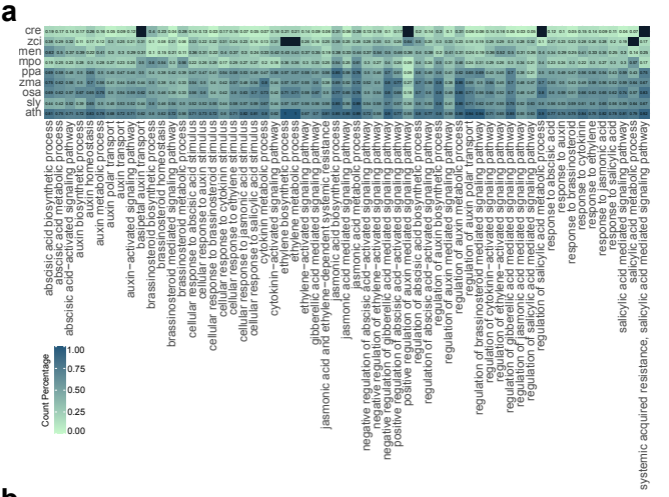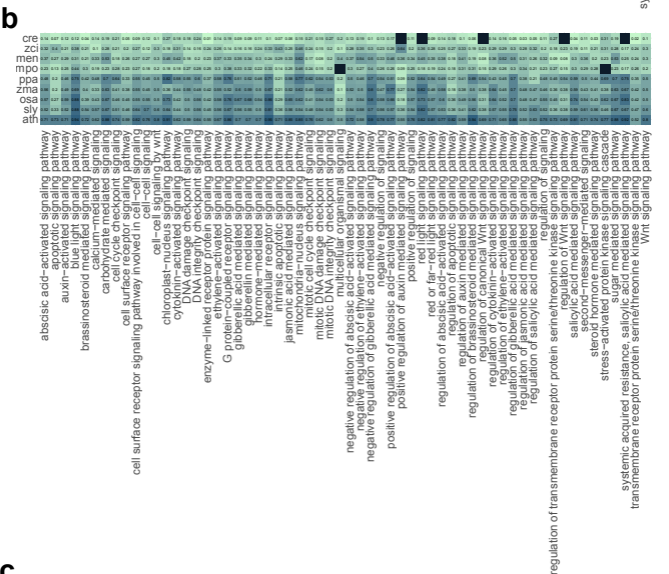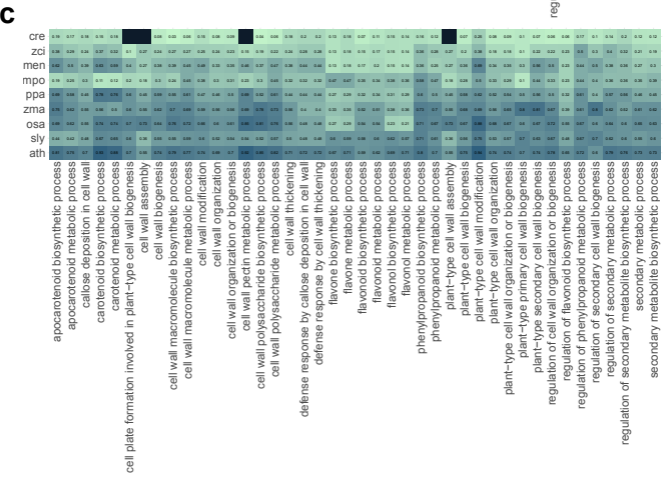

Supplement: Supplementary file 3 — Figure S2. The percentage of genes with Gene Ontology (GO) terms related to stress across species. Panels show GO terms related to (a) phytohormone, (b) cell signaling, (c) specialized metabolism and cell wall biogenesis. Assignments to the categories (a–c) were carried out based on relevant keywords; redundancy between GO terms in the category is to be expected because phytohormone biology will also be categorized in cell signaling and a specialized metabolism. Species abbreviations: ath, Arabidopsis thaliana; cre, Chlamydomonas reinhardtii; men, Mesotaenium endlicherianum; mpo, Marchantia polymorpha; osa, Oryza sativa; ppa, Physcomitrium patens; sly, Solanum lycopersicum; zci, Zygnema circumcarinatum; zma, Zea mays. [file TPJ-122-0-s003.pdf]
