# Supplementary material for: Algal origins of core land plant stress response subnetworks
Source: Plant J. 2025 Jun 23;122(6):e70291. doi: 10.1111/tpj.70291 (PMC12185132; doi:10.1111/tpj.70291)
Supplement: Supplementary file 2 — Figure S1. Cases of Arabidopsis thaliana and Physcomitrium patens networks that highlight the prediction of characterized concertedly acting genes. Two examples of stress response networks obtained from our approach. The network computed for the responses to UV in A. thaliana: (a) enriched GO terms and (b) gene neighborhoods. The network computed for the responses to jasmonate and wounding: (c) enriched GO terms and (d) gene neighborhoods; zoom in highlight interesting modules, showcasing the procedure in the interactive online tool. [file TPJ-122-0-s004.pdf]

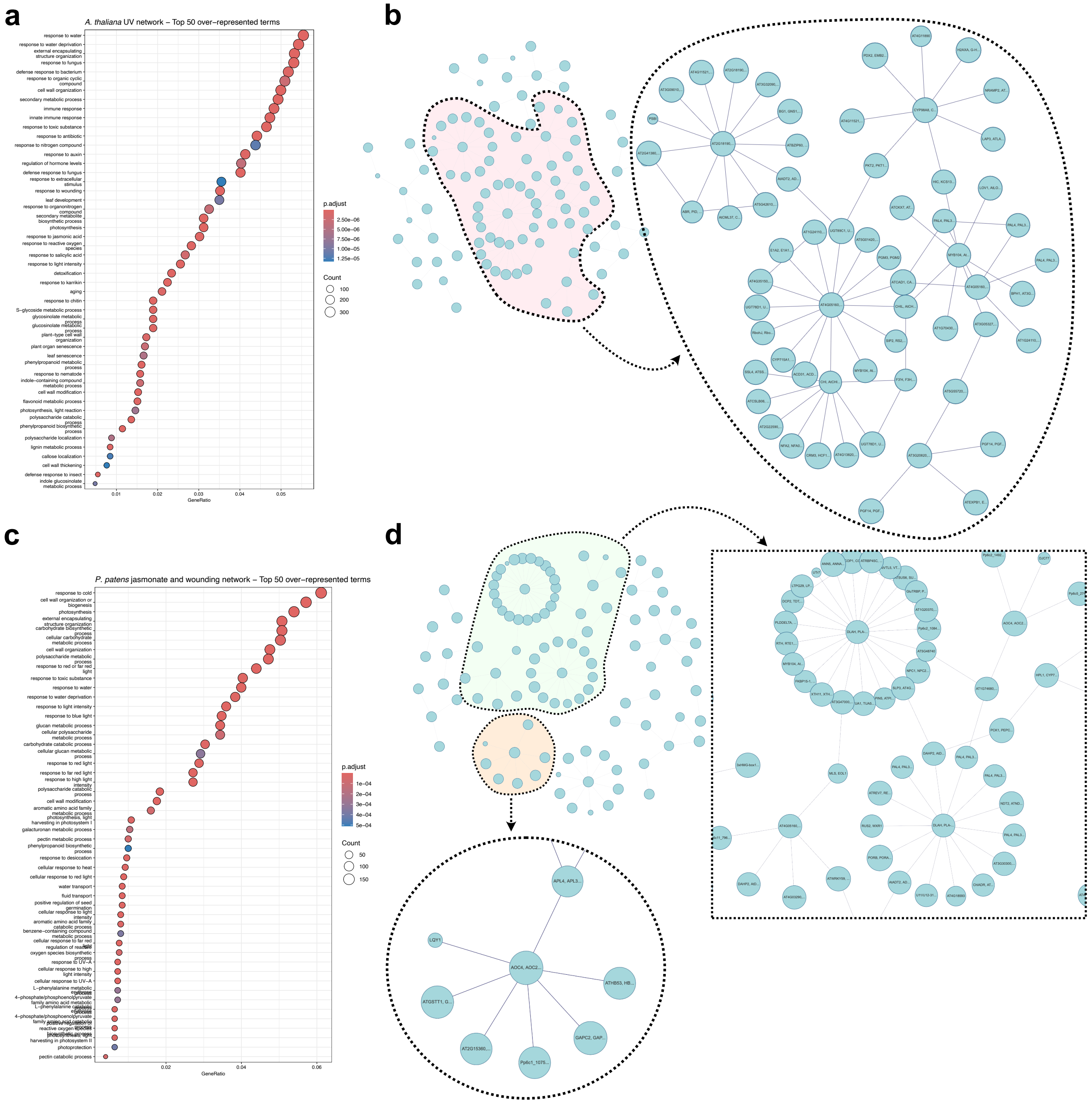
a
A. thaliana UV network – Top 50 over-represented terms
response to water
response to water deprivation
external encapsulating structure organization
response to fungus
defense response to bacterium
response to organic cyclic compound
cell wall organization
secondary metabolic process
immune response
innate immune response
response to toxic substance
response to antibiotic
response to nitrogen compound
response to auxin
regulation of hormone levels
defense response to fungus
response to extracellular stimulus
response to wounding
leaf development
response to organonitrogen compound
secondary metabolite biosynthetic process
photosynthesis
response to jasmonic acid
response to reactive oxygen species
response to salicylic acid
response to light intensity
detoxification
response to karrikin
aging
response to chitin
S-glycoside metabolic process
glycosinolate metabolic process
glucosinolate metabolic process
plant-type cell wall organization
plant organ senescence
leaf senescence
phenylpropanoid metabolic process
response to nematode
indole-containing compound metabolic process
cell wall modification
flavonoid metabolic process
photosynthesis, light reaction
polysaccharide catabolic process
phenylpropanoid biosynthetic process
polysaccharide localization
lignin metabolic process
callose localization
cell wall thickening
defense response to insect
indole glucosinolate metabolic process
0.01
0.02
0.03
0.04
0.05
GeneRatio
p.adjust
2.50e-06
5.00e-06
7.50e-06
1.00e-05
1.25e-05
Count
100
200
300
b
c
P. patens jasmonate and wounding network – Top 50 over-represented terms
response to cold
cell wall organization or biogenesis
photosynthesis
external encapsulating structure organization
carbohydrate biosynthetic process
cellular carbohydrate metabolic process
cell wall organization
polysaccharide metabolic process
response to red or far red light
response to toxic substance
response to water
response to water deprivation
response to light intensity
response to blue light
glucan metabolic process
cellular polysaccharide metabolic process
carbohydrate catabolic process
cellular glucan metabolic process
response to red light
response to far red light
response to high light intensity
polysaccharide catabolic process
cell wall modification
aromatic amino acid family metabolic process
photosynthesis, light harvesting in photosystem I
galacturonan metabolic process
pectin metabolic process
phenylpropanoid biosynthetic process
response to desiccation
cellular response to heat
cellular response to red light
water transport
fluid transport
positive regulation of seed germination
cellular response to light intensity
aromatic amino acid family catabolic process
benzene-containing compound metabolic process
cellular response to far red light
regulation of reactive oxygen species biosynthetic process
response to UV-A
cellular response to high light intensity
cellular response to UV-A
L-phenylalanine metabolic process
4-phosphate/phosphoenolpyruvate family amino acid metabolic process
L-phenylalanine catabolic process
4-phosphate/phosphoenolpyruvate family amino acid catabolic process
positive regulation of reactive oxygen species biosynthetic process
photosynthesis, light harvesting in photosystem II
photoprotection
pectin catabolic process
0.02
0.04
0.06
GeneRatio
p.adjust
1e-04
2e-04
3e-04
4e-04
5e-04
Count
50
100
150
d
APL4, APL3...
LQY1
AOC4, AOC2...
ATHB53, HB...
ATGSTT1, G...
AT2G15360,...
Pp6c1_1075...
GAPC2, GAP...
